# Comparison of Cytotoxic Activity of Anticancer Drugs against Various Human Tumor Cell Lines Using *In Vitro* Cell-Based Approach

**Published:** 2012-03

**Authors:** Leila Florento, Ronald Matias, Elena Tuaño, Katherine Santiago, Frederick dela Cruz, Alexander Tuazon

**Affiliations:** *Department of Biological Sciences, Medical and Regulatory Affairs Division, United Laboratories, Inc., 66 United St. Mandaluyong City, Philippines*

**Keywords:** IC_50_, *in vitro* cytotoxicity, dose-response, relative potency

## Abstract

Chemotherapy is the main treatment modality for certain types of cancer. It is important to monitor and ensure that these chemotherapeutic drugs are potent and effective prior to patient administration. The objective of the study is to evaluate the cytotoxic activity and potency of selected commercially available generic anticancer drugs in comparison with originator using various human cancer cell lines in an *in vitro* cell-based assay. Half-maximal inhibitory concentration (IC_50_) of the different chemotherapeutic agents was obtained from an experimentally derived dose-response curve. Relative potency of the drugs was estimated according to Parallel Line assay. This study demonstrated that the selected generic oncology products tested had similar efficacy compared with the originator. Both products showed comparable results as shown both *in vitro* cytotoxicity assay and statistical analysis. *In vitro* cell-based cytotoxicity assay promises to be a useful, reliable and rapid method for demonstrating chemotherapeutic drug activity.

## INTRODUCTION

Chemotherapy with cytotoxic drugs is the main treatment modality for certain types of cancer (1). It is important to monitor and ensure that these chemotherapeutic drugs are potent and effective prior to patient administration. *In vitro* cell-based assays have been developed to rapidly determine the cytotoxic activity of several compounds. Cell-based assays are also useful in identifying variations in susceptibility of different target cells to several chemotherapeutic agents (2, 3).

There are several chemotherapeutic drugs available in the market. Potency of these products has been tested at the site of production and has passed quality control and quality assurance requirements. However, these products may be exposed to different environmental stress conditions during transport and storage. Hence, it may be necessary to test randomly selected lots for their activity to ensure efficacy.

The objective of the study is to evaluate the cytotoxic activity and potency of selected commercially available generic anticancer drugs in comparison with originator using various human cancer cell lines in an *in vitro* cell-based assay.

## MATERIALS AND METHODS

### Cell lines

HT-29 and HeLa cells were grown on E-MEM Minimum Essential Medium (MEM) with Earle’s salt and nonessential amino acids, supplemented with 5% heat-inactivated fetal calf serum (FCS), 1 mM sodium pyruvate, and 2 mM L-glutamate. NCI-H2126 cells were grown on HITES medium supplemented with 5% fetal bovine, 0.005 mg/mL insulin, 0.01 mg/mL transferrin, 30 nM sodium selenite, 10 nM hydrocortisone, 10 nM beta-estradiol, 10 mM HEPES, and 2 mM L-glutamine. SKOV-3 cells were grown on McCoy’s 5a medium supplemented with 10% fetal bovine serum. PC-3 cells were grown on F12-K medium supplemented with 10% fetal bovine serum. All cells were grown at 37°C in 95% air with the addition of 5% CO_2_.

### Cytotoxicity Assay

**MTT Assay.** The details of this assay have been described previously (3, 4). Briefly, cells were seeded at 1 × 10^5^ cells/mL in 96 well microtiter plates in Minimum Essential Medium with fetal bovine serum. The cells were incubated overnight for attachment. Drug concentrations in serial three-fold dilutions were added in triplicates and incubated for 48h at 5% CO_2_ at 37°C (see list of drugs and corresponding cell line used in Table 1). Thereafter, the cells were treated with 3-[4,5-dimethylthiazol-2-yl]-2,5-diphenyltratrazolium bromide (MTT) (Sigma Chemical Co., St. Louis, MO). Four hours later, all of the medium including MTT solution (5 mg/mL) was aspirated from the wells. The remaining formazan crystals were dissolved in DMSO and the absorbance was measured at 570 nm using a 96 well microplate reader (Synergy^TM^ HT, Bio-Tek Instruments, Inc.). The cytotoxicity index was determined using the untreated cells as negative control. The percentage of cytotoxicity was calculated using the background-corrected absorbance follows (3, 4): % cytotoxicity=1−absorbance of experimental wellabsorbance of negative control well×100

**Table 1:** List of Generic Oncology Products, the innovator products and the corresponding tumor cell line used

Generic-oncology Products	Originator	Tumor cell line used	Origin

Paclitaxel	Brand X	MCF-7	Breast carcinoma
		NCI-H2126	non-small cell lung carcinoma
Docetaxel	Brand X	MCF-7	Breast carcinoma
		SKOV-3	ovarian carcinoma
		PC-3	prostate carcinoma
		NCI-H2126	non-small cell lung carcinoma
Oxaliplatin	Brand X	HT-29	colorectal carcinoma
Bicalutamide	Brand X	PC-3	prostate carcinoma
Anastrozole	Brand X	MCF-7	breast carcinoma

### IC_50_ determination

The IC_50_ was extrapolated from the dose-response graph. The drug concentration that reduced the viability of cells by 50% (IC_50_) was determined by plotting triplicate data points over a concentration range and calculating values using regression analysis of PRISM program.

### Statistical Analysis

Analysis of the dose-response curve was done using the Software GraphPad PRISM. Relative potency of the drugs was estimated according to Parallel Line Assay (*PLA version 1.2.06*) (5, 6). Calculation of confidence limits and significance testing were made at the level of *p*=0.05.

## RESULTS

### Evaluation of Cytotoxic Effect Using MTT Test

Metabolic activity can be evaluated by measuring the activity of a mitochondrial enzyme succinate dehydrogenase using MTT test. MTT is designed for the quantification of cytotoxic index in cell population using 96 well plate format. This test is widely used in the *in vitro* evaluation of the cytotoxic potency of drugs. In the present study we applied the MTT test to evaluate the potency of selected commercially available generic anticancer drugs in comparison with originator using various human cancer cell lines in an *in vitro* cell-based assay.

### Dose-response curve

**Figure 1:**
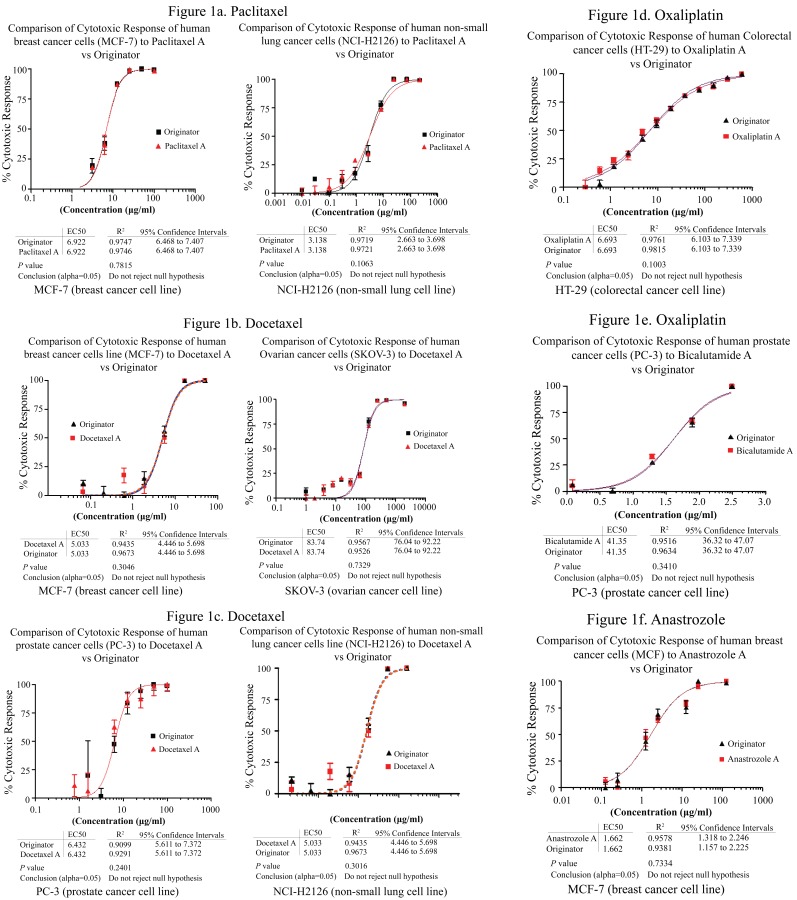
Comparison of the dose-response curve of selected generic oncology products with originator. A similar dose-response was observed in increasing dose concentration of the drugs added to the cells in culture. The dose-response is similar and statistical analysis proved that the difference is not significant (*p*>0.05). The IC_50_ was estimated from the curve generated. The lower the IC_50_, the more cytotoxic the drug is to that specific cancer cell type.

The cytotoxic response of the different cell lines to different generic oncology products versus the originator is shown in Figure 1 (a-f). The dose response curve exhibited by the generic oncology products is comparable with the originator using the indicated cancer cell lines.

### IC_50_ and Relative Potency

Table 2 shows the half maximal effective dose deduced from the generated dose-response curve and the relative potency of the selected generic oncology products. Statistical analysis was done using parallel line assay (*PLA version 1.2.06*) and both were found to be comparably cytotoxic and potent. A value of 1 (± 0.2) means that the relative potency of the tested product is almost the same as that of the competitor product (6). The generic oncology products in comparison with the competitor passed the test for linearity, test of slope and test of parallelism.

**Table 2:** The half maximal effective dose (IC_50_) and relative potency of the selected generic oncology products

CELL LINES	IC_50_ (m/mL) 95% Confidence Interval	Relative Potency 95% Confidence Interval

PACLITAXEL		
MCF-7 (breast cancer cells)	6.9 (6.19-7.58)	0.9 (0.72-1.15)
NCI-H2126 (non-small cell lung cells)	3.1 (2.66-3.69)	0.95 (0.45-1.94)
DOCETAXEL		
MCF-7 (breast cancer cells)	5 (4.44-5.69)	1.2 (0.69-2.15)
SKOV-3 (ovarian cancer cells)	83.7 (76.04-92.2)	1.08 (0.65-1.78)
PC-3 (prostate cancer cells)	6.4 (5.61-7.37)	0.9 (0.48-1.53)
NCI-H2126 (non-small cell lung cells)	5 (4.44-5.69)	1.1 (0.72-1.79)
OXALIPLATIN		
HT-29 (colorectal cancer cells)	6.7 (6.10-7.33)	0.9 (0.71-1.01)
BICALUTAMIDE		
PC-3 (prostate cancer cells)	41.3 (36.3-47.07)	1.1 (0.97-1.3)
ANASTROZOLE		
MCF-7 (breast cancer cells)	1.6 (1.31-2.24)	0.9 (0.45-1.96)

## DISCUSSION

We used a cell-based assay to demonstrate that the potency of the generic oncology products is comparable with innovator drugs. Drug chemosensitivity assays were developed to evaluate anti-neoplastic drugs using cell cultures. Incorporation of cell culture studies offers good possibility as gold standard to assess the drugs due to the controlled conditions and easy procedures. A cytotoxicity test based on mitochondrial activity is then used to evaluate *in vitro* drug efficacy (4).

One of the major goals of oncology is to predict the response of patients with cancer to chemotherapeutic agents by employing laboratory methods variously called “tumor chemosensitivity assays”, “drug response assays”, or “drug sensitivity assays”, *in vitro* (4). The MTT assay is one of the methods used to predict the drug response in malignancies (4). *In vitro* cytotoxicity assays were applied to evaluate anti-neoplastic drugs on target cell lines. Incorporation of cell culture studies offers good possibility as gold standard to assess the drugs due to the controlled conditions and automated procedures (6).

The cytotoxic response of different cell lines to different oncology products is evaluated using high-throughput cell-based assay, the MTT assay. MTT assay is a laboratory test and a standard colorimetric assay (an assay which measures changes in colour) for measuring cellular proliferation (cell growth) (7, 8). The MTT assay reported by Mosmann is a rapid and convenient colorimetric assay for cellular growth and survival *in vitro*. In this paper, the MTT assay was modified as a chemosensitivity test, and its potential was investigated. This method also has several advantages with respect to rapidity, quantitation, management of many samples, and cell number required for the assay. Application of this assay to chemosensitivity testing seems to be valuable and useful.

MTT measures cell respiration and the amount of formazan produced is proportional to the number of living cells present in culture. An increase or decrease in cell number results in a concomitant change in the amount of formazan formed, indicating the degree of cytotoxicity caused by the drug. IC_50_ is the concentration of the tested drug able to cause the death of 50% of the cells and can be predictive of the degree of cytotoxic effect. The lower the value, the more cytotoxic is the substance. Figure 1 (a-f) shows the comparison of the IC_50_ of some chemotherapeutic drugs against human cancer cell lines.

The MTT-based assay relies upon the cellular reduction of tetrazolium salts to their intensely colored formazans. The test is easy to perform in hematological malignancies and is adaptable for high throughput of samples, although there are some minor limitations in its application resulting from metabolic interference. This class of assay is highly accurate for predicting drug resistance, whereas its predictive value for drug sensitivity depends on the type of disease and drug or drug combination used (9). They have been found to predict clinical response to fluradabine FLD in B-CLL and were useful for predetermining clinical potential of a single drug or drug combination in AML patients (4). The premise of *in vitro* drug response testing is that it can provide the knowledge of the relative efficacy of the various agents used in standard therapy before an empiric in vivo trial. Cell-based assays may help in the selection of chemotherapeutic drugs with the greatest likelihood for clinical effectiveness, and in the exclusion of ineffective therapy. This can lead to improved disease management, response, survival and use of financial resources. This study demonstrated that selected generic oncology products tested has similar efficacy compared with originator. Both products showed comparable results as proven by both *in vitro* cytotoxicity assay and statistical analysis.
